# Unraveling neurovascular mysteries: the role of endothelial glycocalyx dysfunction in Alzheimer’s disease pathogenesis

**DOI:** 10.3389/fphys.2024.1394725

**Published:** 2024-07-04

**Authors:** Nicholas O’Hare, Karina Millican, Eno E. Ebong

**Affiliations:** ^1^ Department of Chemical Engineering, Northeastern University, Boston, MA, United States; ^2^ Department of Bioengineering, Northeastern University, Boston, MA, United States; ^3^ Department of Neuroscience, Albert Einstein College of Medicine, New York, NY, United States

**Keywords:** endothelial glycocalyx, Alzheimer’s disease, blood-brain barrier, neurovascular dysfunction, vascular mechanobiology, vascular etiology

## Abstract

While cardiovascular disease, cancer, and human immunodeficiency virus (HIV) mortality rates have decreased over the past 20 years, Alzheimer’s Disease (AD) deaths have risen by 145% since 2010. Despite significant research efforts, effective AD treatments remain elusive due to a poorly defined etiology and difficulty in targeting events that occur too downstream of disease onset. In hopes of elucidating alternative treatment pathways, now, AD is commonly being more broadly defined not only as a neurological disorder but also as a progression of a variety of cerebrovascular pathologies highlighted by the breakdown of the blood-brain barrier. The endothelial glycocalyx (GCX), which is an essential regulator of vascular physiology, plays a crucial role in the function of the neurovascular system, acting as an essential vascular mechanotransducer to facilitate ultimate blood-brain homeostasis. Shedding of the cerebrovascular GCX could be an early indication of neurovascular dysfunction and may subsequently progress neurodegenerative diseases like AD. Recent advances in in vitro modeling, gene/protein silencing, and imaging techniques offer new avenues of scrutinizing the GCX’s effects on AD-related neurovascular pathology. Initial studies indicate GCX degradation in AD and other neurodegenerative diseases and have begun to demonstrate a possible link to GCX loss and cerebrovascular dysfunction. This review will scrutinize the GCX’s contribution to known vascular etiologies of AD and propose future work aimed at continuing to uncover the relationship between GCX dysfunction and eventual AD-associated neurological deterioration.

## 1 Introduction: the role of vascular dysfunction in Alzheimer’s disease

Alzheimer’s disease (AD) is a neurodegenerative disorder that causes a gradual and irreversible loss of neuronal function in the brain, resulting in progressive impairment of cognitive abilities, such as memory, decision-making, and communication skills (Sheppard and Coleman, 2020). As the disease advances, it leads to increasingly severe symptoms and eventual loss of independence, leading to devastating effects not only for the individual but also their loved ones, caregivers, and society as a whole. In the United States alone, an estimated 6.2 million people over the age of 65 suffer from the condition as of 2021 (Alzheimer, 2021). Unfortunately, the number of cases of AD is projected to increase two to four times by 2050 without significant medical breakthroughs (Alzheimer's disease facts and figures, 2021; Kumar et al., 2022). There are currently no cures for AD, and the only clinical successes have been related to delaying the inevitable cognitive and behavioral decline associated with the disease.

Effective treatments for AD remain elusive due to the complex and poorly understood etiology of the disorder. Traditionally, Amyloid beta (Aβ) plaques and neurofibrillary tangles (NFTs) are considered the hallmark pathological features of AD (De-Paula et al., 2012). However, targeting these protein aggregates has been challenging, and clinical trials focused on these deposits have shown mixed results, suggesting other mechanisms contribute to the development and progression of AD (Braak and Braak, 1995; Korczyn, 2008; Korczyn, 2012). Thus, it is imperative to investigate additional disease factors for better treatments. This includes exploring the vascular hypothesis of AD, which could significantly advance research and therapies.

Redefining AD as not only a neurological disorder, but rather a combination of vascular abnormalities and neurodegeneration could provide a novel perspective on disease pathology. Despite AD being traditionally considered a non-vascular dementia, virtually all AD patients exhibit impaired vascular function in their brains (De La Torre, 2002). Although this correlation between vascular disease and AD was proposed nearly 30 years ago, the underlying link between vascular dysfunction and AD progression remains relatively understudied (de la Torre and Mussivand, 1993). Epidemiological studies indicate that neurovascular dysfunction often precedes conventional neuro-centric pathology in AD patients, offering a promising avenue for novel treatment of AD (Prohovnik et al., 1988; Ruitenberg et al., 2005; Li et al., 2010). This observation is critical to supporting the notion that not only do vascular abnormalities coincide with AD, but they may be promoting the accumulation of toxic protein aggregates in the brain, upstream of symptom manifestation. Future work aims to identify the causal impact of vascular dysfunction to AD symptoms and clarify the weight of these vascular events.

The vascular hypothesis proposes that AD development may originate from neurovascular unit (NVU) breakdown, leading to significant neuronal health changes characteristic of AD. This dysfunction is driven by various interconnected neurovascular pathologies including blood-brain barrier (BBB) breakdown, neurovascular decoupling, and endothelial dysfunction. Structurally, the BBB plays an essential role in facilitating the transport of nutrients and waste products in and out of the brain’s fragile environment (Hussain et al., 2021). When the BBB is damaged, it allows for neuroinflammatory compounds to enter while hindering the clearance of waste products such as AD-associated protein precursors. This leads to protein aggregation, glial inflammation, and neuronal death (Chen and Strickland, 1997; Mhatre et al., 2004; Schachtrup et al., 2007; Sweeney et al., 2018). Congruently, neurovascular decoupling, the process in which the vasculature cannot adequately adapt to the brain’s demands, also promotes AD manifestation through inadequate nutrient delivery and impeded waste clearance (Zhu et al., 2022). Finally, endothelial dysfunction within the brain microvasculature induces oxidative stress and cellular inflammation while simultaneously further deteriorating BBB integrity and the responsiveness of blood vessels (Di Marco et al., 2015). All these vascular pathologies have been observed in patients presenting early-stage AD cognitive dysfunction, suggesting their contribution to disease progression (Di Marco et al., 2015; Montagne et al., 2015; van de Haar et al., 2016). Further investigating the impact of these pathologies to AD progression and uncovering the initial insult which causes cerebral vascular dysfunction could dramatically alter treatment approaches to AD.

**FIGURE 1:**
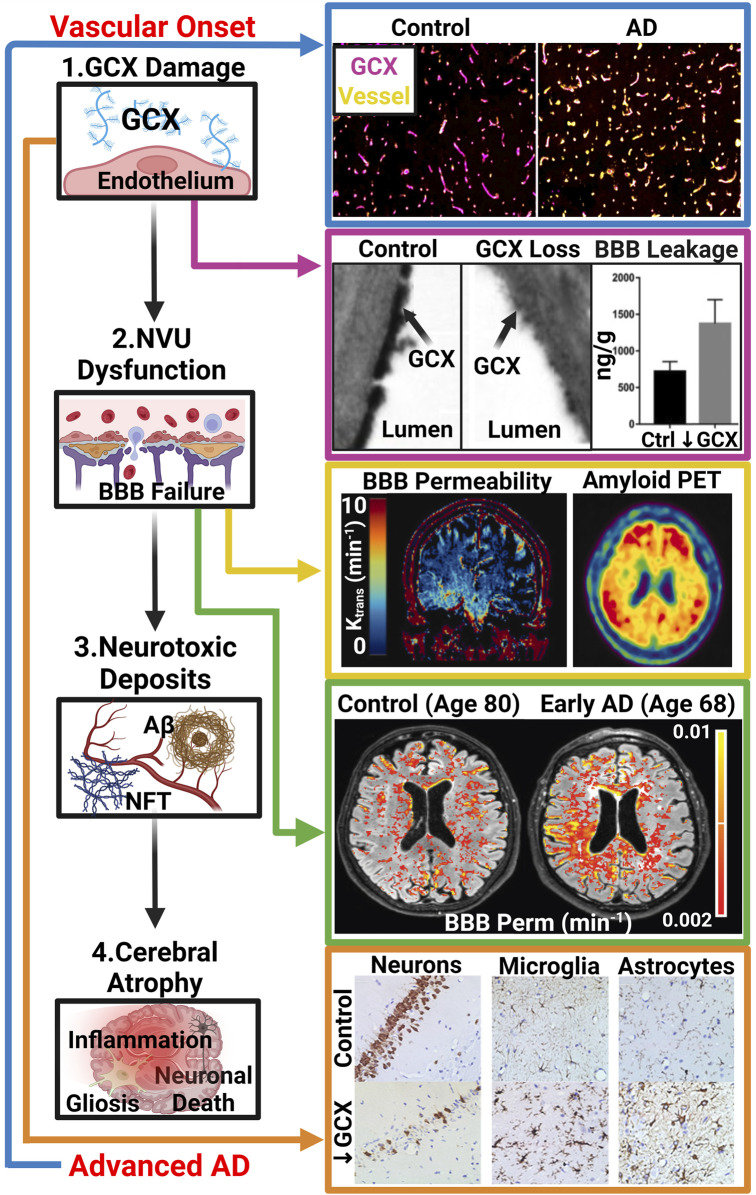
The GCX-Vascular Hypothesis of AD: Hypothesized sequence of events linking GCX degradation to AD pathology: (1.) Various vascular insults induce damage to the GCX. (2.) GCX damage initiates NVU dysfunction, notably affecting the BBB. (3.) NVU dysregulation leads to abnormal accumulation of neurotoxic deposits including Aβ and NFTs within the brain, characteristic of AD. (4.) Subsequently, these protein aggregates induce brain inflammation, gliosis, and neuronal death, facilitating cognitive decline. Several recent studies have supported this GCX-rooted hypothesis. (Blue Arrow): Smyth et al. observed a significant reduction in GCX integrity in post-mortem AD tissue compared to healthy controls, suggesting a correlation between late-stage AD and GCX integrity via immunostaining of UEA1 lectin for GCX and Collagen type IV for cerebral vasculature. Adapted from Figure 5 in Smyth LCD, et al. Acta Neuropathologica Communications. 2022; 10(1), Copyright (2022) by Acta Neuropathologica Communications (Smyth et al., 2022). This figure is sourced from an open-access publication. (Magenta Arrow): Zhu et al. demonstrated increased leakage of Evan’s blue dye into the brain parenchyma upon GCX degradation using hyaluronidase treatment in mice, indicating GCX loss triggers NVU dysfunction. Adapted from Figure 2 in Zhu J., et al., J Cereb Blood Flow Metab. 2018; 38(11):1979-92, Copyright (2018) by the Journal of Cerebral Blood Flow (32). Used with permission. (Yellow Arrow): Moon et al. showed a regional correlation between increased BBB permeability and AD protein aggregation in early AD patients using DCE MRI and amyloid positron emission tomography. Adapted from Figure 1 in Moon Y., et al. J Cereb Blood Flow Metab. 2023; 43(11):1813-25, Copyright (2023) by the Journal of Cerebral Blood Flow ((Moon et al., 2023)). Used with permission. (Green Arrow): Furthermore, van de Haar et al. observed global increases of BBB permeability in patients suffering from early cognitive decline associated with AD compared to controls independent of age. Adapted from van de Haar HJ, et al. Radiology. 2016; 281(2):527-35, Copyright (2016) by the Journal of Radiology ((van de Haar et al., 2016)). Used with Permission. (Orange Arrow) Finally, Zhu et al. investigated the impact of GCX degradation on neuronal loss and glial cell activation through immunostaining of neurons (NeuN), microglia (Iba1), and astrocytes (GFAP), revealing significant reductions in neuron count and activated glial cells resembling cerebral pathologies in AD. Adapted from Figure 5 in Zhu J., et al. J Cereb Blood Flow Metab. 2018; 38(11):1979-92, Copyright (2018) by the Journal of Cerebral Blood Flow ((Zhu et al., 2018)). Used with permission. While these studies support the proposed cascade, further research is needed to elucidate whether GCX damage is a causative factor in AD manifestation or a consequence of an already pathological state (De La Torre, 2002; van de Haar et al., 2016; Zhu et al., 2018; Govindpani et al., 2019; Zhao et al., 2021; Smyth et al., 2022; Moon et al., 2023). Created with BioRender.com.

Although a vascular role appears likely in the pathogenesis of AD, determining a therapeutic target to mitigate or reverse these vascular pathologies remains an unresolved question. Some of the structures at the neurovascular interface under investigation include tight junctions, intracellular transporters, nitric oxide (NO) regulators, and adhesion molecules (Govindpani et al., 2019; Soto-Rojas et al., 2021). However, as of now, no specific blood-vessel based target which significantly modifies the progression of AD has been identified. Thus, in this review, the focus will be drawn to the vascular endothelial glycocalyx (GCX), a relatively understudied structure which stands as a potential master regulator of neurovascular health. The GCX is a sugar-rich nano-structure that resembles a dense bush and lines the inner part of blood vessels (Reitsma et al., 2007). Due to its diverse functionality and structural diversity, it may play a role in regulating several vascular functions that are disrupted in AD, such as stabilizing vessel permeability, promoting neurovascular coupling, and maintaining physiological redox of the endothelium (Curry and Adamson, 2012; Yen et al., 2015; Qu et al., 2021). Research has already demonstrated the significant role of GCX health in preventing various vascular disorders in the body, and recent discoveries regarding its therapeutic potential are rapidly multiplying (Tarbell and Cancel, 2016; Dogné and Flamion, 2020; Zhao et al., 2021). Therefore here, we will present the current knowledge regarding the GCX and its connection to neurovascular health in the context of AD while also outlining future research directions aimed at unraveling its role in disease pathology. Overall, we will propose a GCX-vascular-hypothesis of AD progression where GCX degradation leads to neurovascular unit dysfunction, ultimately promoting abnormal protein accumulation in the brain resulting in AD manifestation (Figure 1). Although still in its early stages and primarily correlational, recent research has notably progressed our comprehension of the connection between cerebral vascular dysfunction and AD, while also shedding light on the involvement of the GCX (Figure 1) (van de Haar et al., 2016; Zhu et al., 2018; Smyth et al., 2022; Moon et al., 2023). However, it remains imperative for future research to determine whether the loss of GCX precedes subsequent AD events and elucidate the underlying mechanisms. This review seeks to consolidate existing knowledge regarding the GCX’s involvement in advancing neurovascular disease and eventual AD pathology, while also proposing a framework for addressing unanswered questions. In light of the limited therapeutic options available for AD, exploring the GCX for its potential as a novel target presents a source of optimism for improving outcomes in this debilitating condition.

## 2 The endothelial glycocalyx: a potential master regulator of neurovascular unit function

### 2.1 What is the cerebral vascular glycocalyx?

The GCX was first visualized by Luft nearly 60 years ago using electron microscopy (Luft, 1966). Over the past decade, the structure has become increasingly recognized as an essential contributor to vascular homeostasis in health and disease for its diverse roles in vessel permeability, mechanotransduction, and cellular signaling (Alphonsus and Rodseth, 2014; Uchimido et al., 2019; Potje et al., 2020). In light of accumulating evidence indicating that GCX damage precedes subsequent vascular pathologies, there is a growing focus on exploring the therapeutic potential of this structure upon regeneration not only for cardiovascular disease but neurological conditions as well (Reed et al., 2019; Yoon et al., 2022). The GCX was only recently considered as an important component of the extended NVU due to its role in regulating neurovascular health (Stanimirovic and Friedman, 2012). The GCX has been implicated in critical processes within the NVU, including BBB regulation, inflammation control, and NO-mediated neurovascular coupling (Reitsma et al., 2007; Bartosch et al., 2017; Kutuzov et al., 2018). Based on the GCX’s role in these processes, deterioration of the structure may be a precursor to previously described vascular etiologies of AD. Not only are all of these vascular abnormalities present in the majority of AD patients, but initial findings have also indicated that the GCX may be compromised prior to downstream neurological events in these disorders (Reed et al., 2019; Dogné and Flamion, 2020; Yoon et al., 2022). Protecting and restoring the GCX may therefore be a promising strategy for preventing or treating neurodegenerative diseases.

Structurally, the GCX is composed of numerous proteoglycans, glycoproteins, and associated glycosaminoglycans (GAGs) (Reitsma et al., 2007; Moore et al., 2021). The major constituents of the GCX include heparan sulfate (HS) with associated core proteins syndecan and glypican, as well as hyaluronic acid (HA) and associated CD44. While these muco-poly-saccharides have been viewed as the main diagnostic markers and mechanistic contributors to GCX function, other complex carbohydrates found at the cell surface including chondroitin sulfate, keratin sulfate, dermatan sulfate, and sialic acid, are also believed to play roles in endothelial function (Potter et al., 2009; Tarbell and Cancel, 2016). Further investigation is needed to fully differentiate the roles and importance of individual GCX components in regulating NVU physiology and deterring downstream AD pathology (Pahakis et al., 2007; Yang and Schmidt, 2013).

The nuances of the cerebrovascular GCX compared to other vascular beds are still currently under investigation. This delayed unravelling can be ascribed to the inherent difficulties of imaging a vascular nanostructure imbedded within the skull. However, recent microscopy advances have allowed for visualization of the structure like never before. Preliminary two-photon light scanning microscopy (TPLSM) in mice has demonstrated a robust arterial and capillary GCX along the cerebral arterial tree with a thickness that varies with vessel type and did not correlate with vessel diameter (Yoon et al., 2017). Interestingly, capillaries exhibited the thickest GCX in relation to vessel diameter perhaps based on the microcirculation being the prominent location of BBB transport. Utilizing another novel imaging modality in side-stream darkfield microscopy (SDF) in human patients, Haeren et al. observed a thicker GCX in cerebral vasculature compared to sublingual vasculature, indicative of the GCX’s heterogeneity throughout the body and its prospective critical role to brain physiology (Haeren et al., 2017; Haeren et al., 2018). While still in its early stages, the ongoing endeavors to differentiate and unravel the intricacies of the cerebrovascular GCX offer promising prospects for future understanding.

### 2.2 Potential role of the endothelial vascular glycocalyx as a blood-brain barrier function controller

Mounting evidence underscores the pivotal role of the GCX in neurovascular regulation. Further understanding of this relationship could prove pivotal in combatting AD and related neurodegenerative disorders. First, the integrity of the GCX is essential to the regulation of vascular flux throughout the body, and its role in BBB permeability is increasingly recognized (Zhao et al., 2021). Traditionally, it was believed that BBB permeability was mainly regulated through tight junctions and adherens junctions, for paracellular transport, and membrane channels, pumps, and carrier proteins, for transcellular transport pathways. However, the importance of a robust GCX is only now being appreciated for its relevance to maintaining overall BBB integrity (Zhu et al., 2018; Yang et al., 2021; Zhao et al., 2021). Research has indicated dramatic increases in vascular permeability upon GCX structure damage, strongly supporting the notion that GCX health not only plays a correlative role but a causal one in regulating overall BBB permeability and thus downstream AD pathology (Reed et al., 2019; Jin et al., 2021).

**FIGURE 2:**
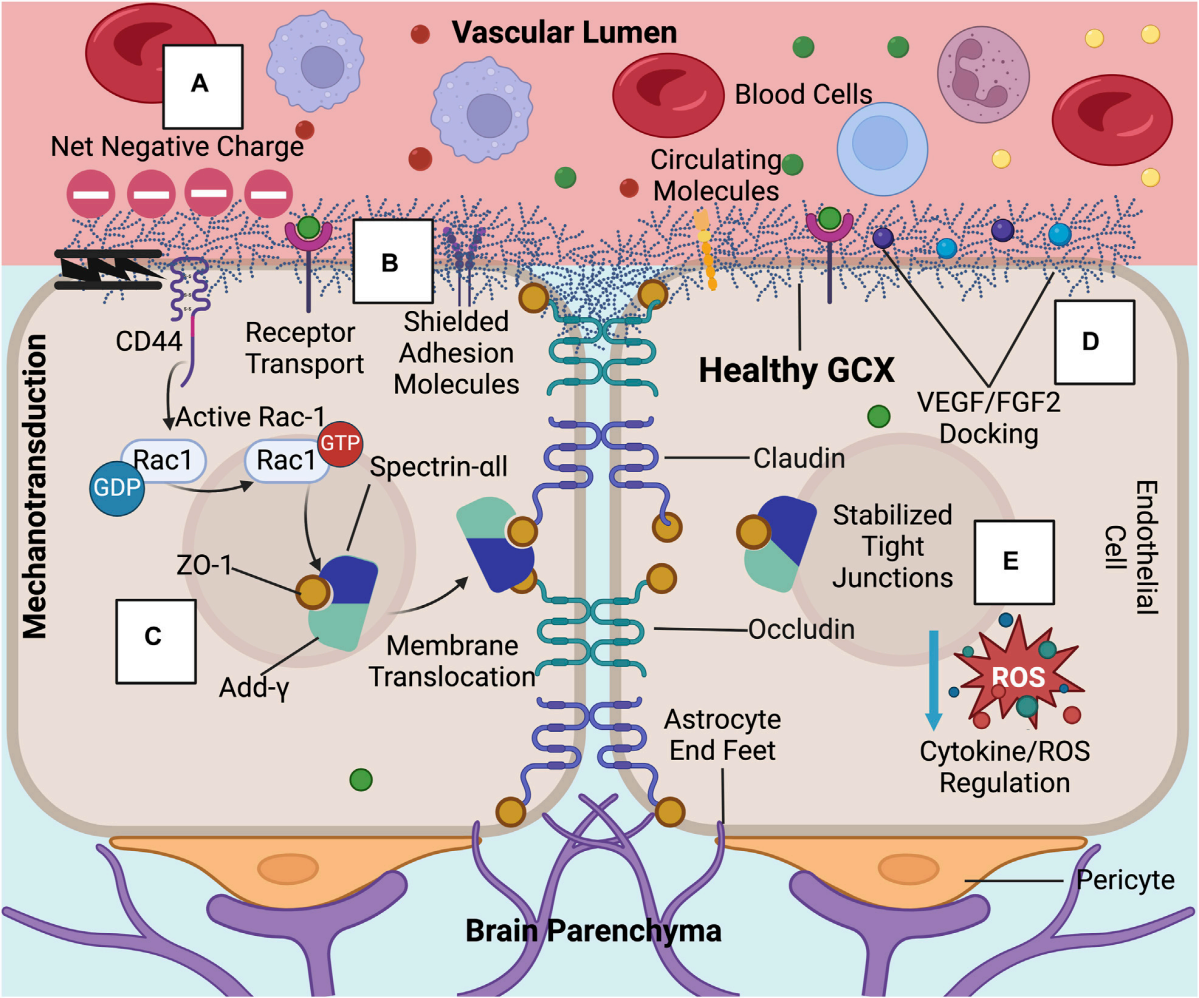
In healthy conditions, the GCX acts as a regulator of permeability and inflammation at the BBB: These GCX specific functions include **(A)** acting as a physical and charge barrier to circulating molecules, **(B)** adhesion molecule shielding, **(C)** stabilizing tight junction complexes and localizing ZO-1 to the cell membrane through mechanotransduction, **(D)** docking permeability inducing factors such as VEGF and FGF2, and **(E)** regulating cytokine and ROS production (Jin et al., 2021; Zhao et al., 2021; Deore et al., 2022). Created with BioRender.com.

**FIGURE 3:**
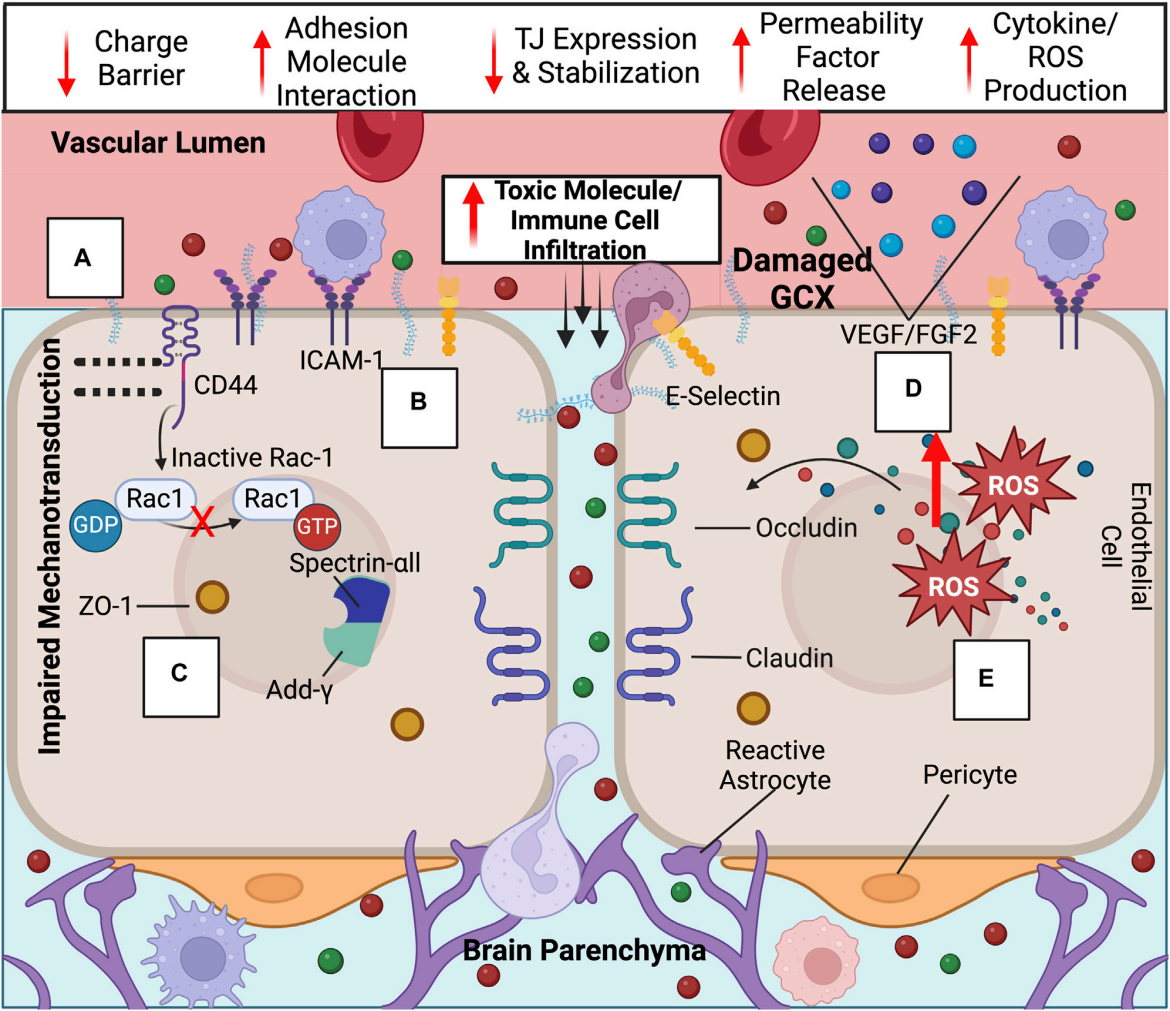
Upon GCX degradation, a variety of transport mechanisms are compromised at the BBB, leading to ultimate increased neurotoxic molecule and immune cell infiltration into the brain: Compromised endothelial functions include the loss of the **(A)** GCX’s negative charge barrier, **(B)** adhesion molecule upregulation and exposure, **(C)** decreased tight junction expression and ZO-1 membrane localization impairment due to failed mechanotransduction, **(D)** permeability factor release, and **(E)** cytokine/ROS production. Created with BioRender.com.

Structurally acting as a physical and charge barrier, the GCX serves as a molecular sieve to the BBB, preventing the entry of neuroinflammatory molecules into the brain while simultaneously allowing essential factors to pass through (Woodcock and Woodcock, 2012; Zhao et al., 2021). The GCX’s role in deterring neurotoxic macromolecules present in the plasma, such as albumin, prothrombin, and plasminogen, may be critical to preventing the progression of AD through deterring the entry of neuroinflammatory molecules (Vink and Duling, 2000; Yang and Schmidt, 2013; Kadry et al., 2020). The GCX also regulates BBB permeability due to the high degree of net negative charge on the GAGs HS and chondroitin sulfate as well as sialic acid sugar chains (Walter et al., 2021) (Figure 2, 3). In enzymatic studies, removal of sialic acid residues on these chains resulted in an increase in albumin infiltration likely due to the reduction in electrostatic resistance (Betteridge et al., 2017). Overall, the GCX’s dual functionality as a physical and charge barrier underscores its significance in regulating molecular access to the central nervous system (CNS).

The GCX plays a crucial role in maintaining the BBB not only through its physical structure but also by influencing cellular signaling which impacts BBB function. It is known to regulate the production and availability of reactive oxygen species (ROS) and cytokines, which are both linked to the disruption of tight junctions such as claudins and occludins at the BBB (Figure 2, 3) (Goes et al., 2001; Rochfort et al., 2014). Additionally, a thinner GCX is associated with reduced expression of crucial transcellular transporters compromised in AD, such as glucose transporter-1 and p-glycoprotein, essential for glucose transport into the brain and Aβ plaque clearance from it, respectively (Gomez González et al., 2011; Winkler et al., 2015; Ding et al., 2021; Santa-Maria et al., 2021). In a study directly examining the effects of GCX impairment on BBB function, Zhu et al. found that hyaluronidase-induced degradation of HA in a rat model led to increased infiltration of Evan’s blue dye into the CNS, reduced tight junction expression, elevated brain water content, and glial activation indicative of neuroinflammation (Figure 1) (Zhu et al., 2018). This highlights the potential involvement of the GCX in BBB permeability regulation and key AD promoting pathologies.

Further research is needed to determine the molecular mechanisms linking GCX loss to BBB disruption and the functions of specific components within the complex, heterogeneous structure. For example, HA removal may trigger a CD44-dependent mechanism of BBB regulation (Al-ahmad et al., 2019). CD44, the primary transmembrane receptor for HA, has been shown by Deore et al. to be critical for BBB formation (Deore et al., 2022). When CD44 is deleted through CRISPR, RAC-1 activation is suppressed whereas RhoA becomes hyperactivated. Consequently, this prevents the initiation of a pathway that involves the recruitment of ZO-1, the actin filament capping protein adducin-ᵞ, and the cytoskeletal protein spectrin-αII to cell-cell junctions, ultimately impairing the integrity of the BBB. (Figures 2, 3). This pathway is dependent on shear-stress-based mechanotransduction, likely due to HA’s role as a mechanosensor (Deore et al., 2022). The interplay of HA, CD44, shear stress, and tight junctions is crucial for maintaining a stable BBB.

Additionally, for HS, which is the most abundant GAG in the GCX, studies show that its enzymatic degradation increases monocyte infiltration into the brain (Floris et al., 2003). HS serves as a docking site for chemokines as well as various other molecules such as fibroblast growth factor 2 (FGF2) and vascular endothelial growth factor (VEGF), all involved in regulating tight junction activity (Figures 2, 3) (Chiodelli et al., 2015; Yang et al., 2021). Furthermore, HS proteoglycans, such as syndecan-1 and glypican-1, may also play critical roles in BBB permeability. Degradation of the GCX enhances syndecan-1 interaction with SRC, promoting caveolae-mediated endocytosis, while glypican-1 loss may reduce Aβ transport out of the brain via complex formation with low-density lipoprotein receptor-related protein 1 (LRP1) (Leite et al., 2020; Zhu et al., 2021).

Ultimately, gaining a deeper understanding of the mechanisms underlying BBB permeability induced by GCX loss and further elucidating the roles of its individual components could provide valuable insights into its relevance to the BBB-based neurodegeneration associated with AD.

### 2.3 Potential role of the endothelial glycocalyx as an inflammation controller for the neurovascular unit

While acting as a BBB permeability regulator, the GCX also serves as a key modulator of neuro- and vascular-inflammation. Alterations in immune cell trafficking and endothelial function may lead to AD-related pathologies including Aβ accumulation, oxidative stress, and neuroinflammation (Govindpani et al., 2019). A feedback loop of neurological and vascular dysfunction results in a severely diseased state of cognitive decline. Could GCX dysfunction precede these downstream events?

In physiological conditions, the GCX plays a crucial role in regulating leukocyte adhesion to the endothelium. The GCX in rat capillaries is believed to range from 200–500 nm, far exceeding the size of endothelial leukocyte receptor and adhesion molecules, such as E-selectin and intercellular adhesion molecule-1 (ICAM-1) (30–40 nm) (van den Berg et al., 2003; Lipowsky, 2012). Due to this size discrepancy, one can logically hypothesize the GCX shields the endothelium from direct interaction with circulating white blood cells (Figures 2, 3). Constantinescu et al. demonstrated that after various heparinase (an enzymatic cleaver of HS) concentrations were administered to mice, the number of adherent leukocytes increased significantly (Constantinescu et al., 2003). Not only does the GCX shield endothelial cells from leukocyte interaction with adhesion molecules, but it may also upregulate adhesion molecule expression upon shedding. In an *in vitro* flow environment, ICAM-1 levels increased by 300% upon enzymatic removal compared to static controls. This increase may be mediated by a shear-induced NF-kβ pathway regulated by GCX components (McDonald et al., 2016).

**FIGURE 4:**
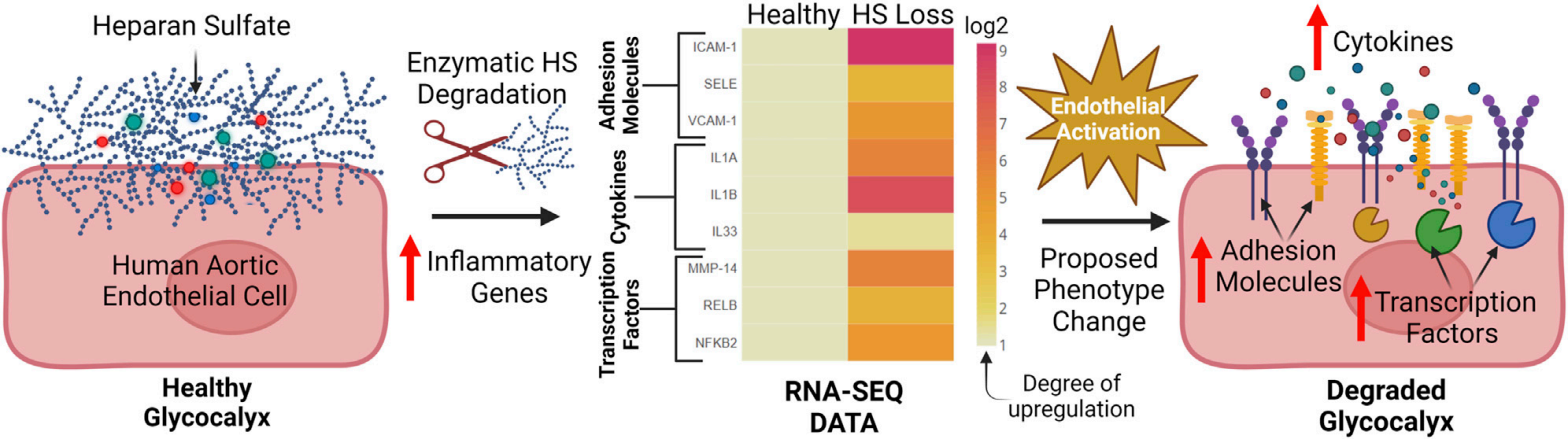
Our lab has found that key inflammatory genes which contribute to endothelial activation are broadly upregulated after HS enzymatic degradation: After enzymatic cleavage of the HS chain on human aortic endothelial cells, 40 out of 60 relevant pro-inflammatory genes involved in endothelial activation were found to be upregulated via RNA-SEQ analysis. These genes include adhesion molecules (ICAM-1, SELE. VCAM-1), cytokines (IL1A, IL1B, IL33), and inflammation promoting transcription factors (MMP-14, RELB, NFKB-2). Data was normalized to the healthy condition (no HS degradation) and displayed through a heat map. This pronounced upregulation of inflammatory markers suggests a shift towards an activated endothelium after HS degradation and possible downstream vascular dysfunction. To the best of our knowledge, no previous studies have holistically interrogated the role of HS in inflammation through RNA-SEQ (Harding et al., 2019a; O Hare et al., 2022). Created with BioRender.com.

In our lab group, we have performed broad RNA-SEQ analysis on human aortic endothelial cells to determine the effects of HS degradation on eliciting endothelial inflammation, a state associated with downstream vascular disease (Figure 4) (Harding et al., 2019a; O Hare et al., 2022). Through analysis of 60 relevant pro-inflammatory markers, 40 of 60 were upregulated upon HS cleavage via Heparinase III treatment. To our knowledge, no study has characterized the effects of HS loss on such a wide range of inflammatory markers implicated in endothelial activation including adhesion molecules, cytokines/chemokines, and pro-inflammatory transcription factors (Figure 4). This work provides further evidence of the pronounced role HS plays in deterring endothelial dysfunction. However, the significance of HS and other GAGs within the GCX for maintaining normal endothelial function in the cerebral vasculature remains underexplored and warrants further investigation to elucidate their roles in mitigating neurovascular dysfunction associated with AD.

### 2.4 Potential role of the endothelial glycocalyx as a facilitator of neurovascular coupling through nitric oxide production

A final key function of the GCX which links it to AD pathology is its role in preventing neurovascular decoupling. Neurovascular decoupling, characterized by an impaired link between neuronal activity and cerebral blood flow, is a prominent vascular pathology in AD (Zhu et al., 2022). Although the brain accounts for only 2% of the body by weight, it demands 15%–20% of the body’s cardiac output, requiring tightly coordinated neurovascular coupling to facilitate its extensive and dynamic nutrient demands (Williams and Leggett, 1989). In AD, there is compelling evidence indicating a notable decrease in essential nutrients reaching the brain and a rise in the accumulation of neurotoxic deposits. This is believed to stem from the vasculature’s inability to meet the brain’s transport demands, thus decoupling the systems (Hock et al., 1997; Park et al., 2005). Furthermore, studies aimed at recovering neurovascular coupling resulted in improved cognitive function in AD mouse models, suggesting a causal link to the disease (Tong et al., 2012; Tarantini et al., 2017). Based on this evidence, proper blood-brain signaling is essential to preventing later-stage AD.

**FIGURE 5:**
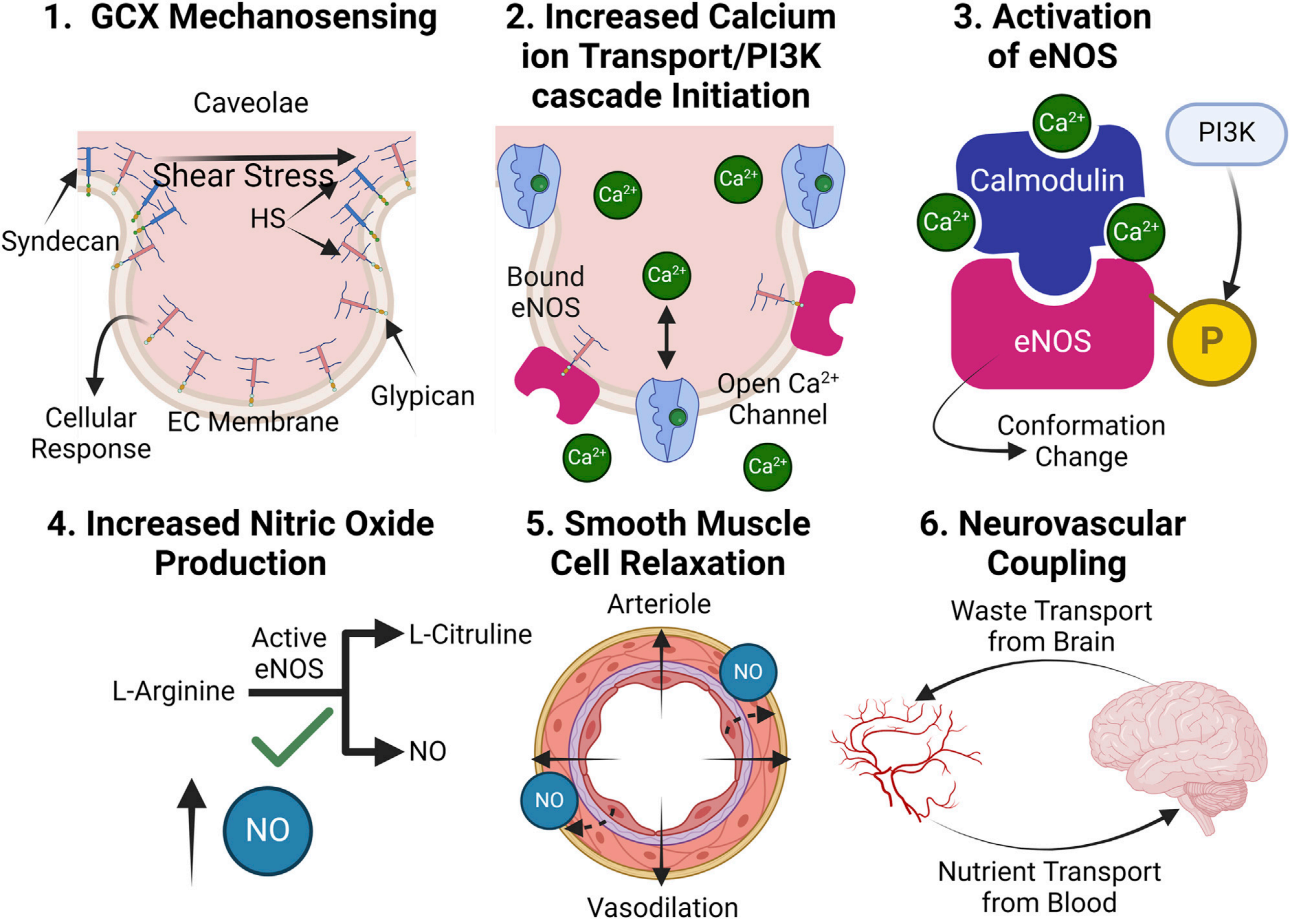
In healthy conditions, the GCX plays an essential role in facilitating proper NO production: (1.) The GCX acts a mechanosensor upon shear stress exposure, facilitating a variety of essential cellular responses. (2.) Such responses include the opening of calcium ion channels, which increases calcium ion availability within the cell and PI3K pathway initiation, leading to eNOS phosphorylation. (3.) Calcium ions form a complex with calmodulin and phosphorylated eNOS, converting inactive eNOS into its active form. (4.) Active eNOS catalytically converts L-arginine into NO and L-citruline. (5.) NO release relaxes smooth muscle cells, leading to blood vessel dilation. (6.) Proper dilation allows for proper blood supply of nutrients to the brain and waste clearance out of it, effectively coupling the two systems (Tarbell and Pahakis, 2006; Dudzinski and Michel, 2007; Kumagai et al., 2009). Created with BioRender.com.

**FIGURE 6:**
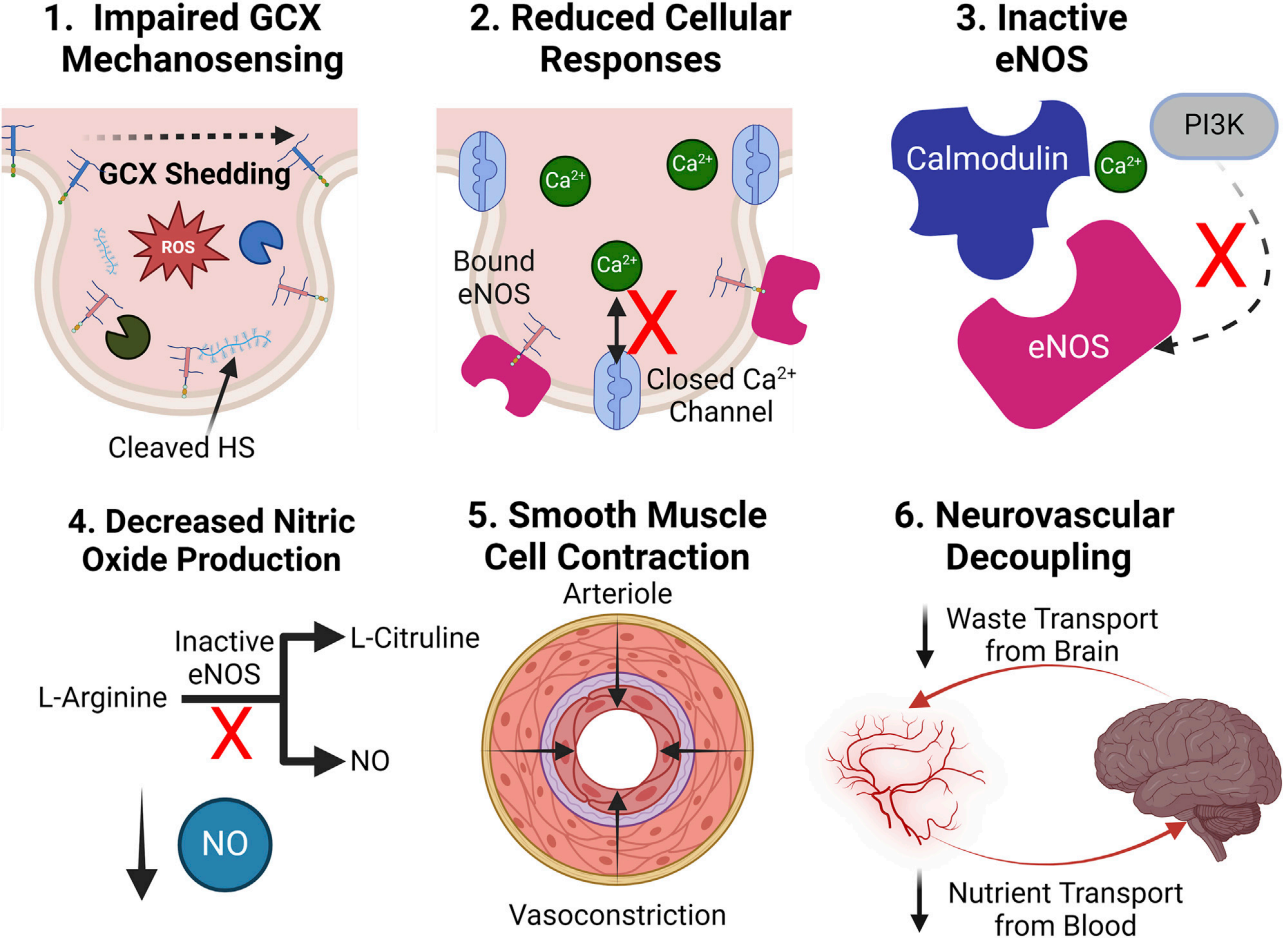
Upon GCX degradation, NO production is impaired, leading to neurovascular decoupling: (1.) Upon degradation, improper mechanotransduction of fluid shear stress stimulus occurs. (2.) Impaired cellular function leaves calcium ion channels closed, and the PI3K pathway inactive. (3.) eNOS fails to conform into its active state. (4.) NO is not generated without an active eNOS catalysis. (5.) With a lack of NO bioavailability, smooth muscle cells contract, restricting blood flow. (6.) A reduction in blood flow causes the nutrient transport to the brain to not meet its demand while waste transport out of the brain slows, leading to toxic accumulations, decoupling the two systems. Created with BioRender.com.

The GCX is an essential contributor to preventing AD-associated neurovascular decoupling through regulating the mechano-physiology response of blood vessels in the cerebral vasculature. In particular, it plays a critical role in NO production, the key molecule responsible for vasodilation and subsequent neurovascular coupling (Yen et al., 2015; Dormanns et al., 2016). Endothelial NO generation relies on GCX mechanotransduction, which is compromised when the GCX is degraded (Figures 5, 6). When the GCX is intact, HS senses shear stress applied to the vascular wall and stimulates its attached core protein, glypican, to facilitate a wide range of cellular responses essential to NO production including the opening of calcium ion channels and the activation of the PI3K pathway (Tarbell and Pahakis, 2006; Kumagai et al., 2009; Ebong et al., 2014; Zeng et al., 2015). Although syndecans and glypicans bind HS, glypicans have demonstrated greater importance to the NO pathways potentially due to their propensity to reside in the caveolae, small invaginations in the cell membrane which associate endothelial nitric oxide synthase (eNOS) (Ebong et al., 2014). With this mechanically transduced increase in Ca^2+^, a calcium-calmodulin complex induces a conformational change to eNOS, while PI3K phosphorylates the enzyme, both processes ultimately facilitating eNOS activation (Figures 5, 6). Activated eNOS produces NO by catalyzing a reaction that converts the amino acid L-arginine and oxygen into L-citrulline and NO. Consequently, vascular tone is regulated through the release of NO, a potent vasodilator, leading to normal neurovascular coupling.

Due to the GCX’s involvement in actuating NO production, studies which degrade HS have demonstrated decreased phosphorylated eNOS and subsequent reduced NO production (Ebong et al., 2014; Yen et al., 2015). Subsequent research has utilized atomic force microscopy pulling of key proteoglycans and GAGs of the GCX to identify HS and glypican-1 as being involved in the production of NO, but not syndecan-1, CD44, and HA (Bartosch et al., 2017; Bartosch et al., 2021). These works highlight the GCX’s involvement in the pathway as well as the diverse functions of different components in the structure.

Further implicating previously mentioned inflammatory pathologies with NO production, the relationship between ROS, NO, and the GCX also mediates vascular dysfunction. During oxidative stress, ROS like O_2_^−^ interact with NO, forming more precarious species like reactive nitrogen species (RNS) and peroxynitrite (Wang et al., 2018; Schenck et al., 2021). Simultaneously, NO bioavailability is reduced, and the GCX is ameliorated by superoxide dismutase (Rubio-Gayosso et al., 2006). The combination of oxidative stress, reduction in NO, and GCX deterioration creates a vicious cycle of vascular dysfunction, ultimately leading to neurovascular decoupling and the progression of AD symptoms. Future work is needed to clarify mechanisms related to GCX mechanosensing, NO production, and ultimate control of neurovascular coupling in the context of AD. However, with the structure’s definitive involvement in the NO pathway, targeted regeneration of the GCX stands as a potential approach for alleviating decoupling-related AD pathology.

## 3 The endothelial glycocalyx: a link to Alzheimer’s disease related vascular pathologies?

### 3.1 Evidence of endothelial glycocalyx degradation in Alzheimer’s disease and other neurodegenerative diseases

Despite an expanding body of work ascertaining the GCX’s role in regulating various vascular functions in AD, the connection between GCX shedding and the progression of AD remains inadequately explored. To establish an argument for the GCX’s role in AD, foundational evidence of GCX deterioration must be demonstrated in these conditions. Currently, only a handful of studies directly examine the GCX in AD progression and other neurological disorders. Smyth et al. may have presented the most clinically-relevant evidence for the correlation between GCX shedding and AD (Figure 1). They found reduced vascular density within AD brain tissue compared to healthy controls but normalized this reduction in blood vessels to GCX abundance, further demonstrating that GCX was reduced in brain tissue regardless of the number of vessels. Their findings also elucidated a role of the GCX in deterring neutrophil attachment (Smyth et al., 2022). Others have identified a mechanism of circulating HA and HS proteoglycans in AD neuropathology as well (van Horssen et al., 2001; Reed et al., 2019; Lorente-Gea et al., 2020). Increased levels of heparinase (cleaver of HS) were found in AD brain tissue, providing another conceivable association between GCX shedding and AD progression (Garcia et al., 2017). Loss of HS likely rearranges the BBB, leading to impaired perivascular clearance and subsequent accumulation of Aβ (Zhang et al., 2021). These published findings provide an indication of GCX involvement in AD, although there is a lack of direct structural measurements of the GCX in AD brain microvasculature as well as mechanistic studies relating GCX loss to AD pathology.

To supplement the few studies that have examined the relationship between GCX function and AD, a growing body of work has demonstrated GCX breakdown in other neurodegenerative disorders with similar vascular pathologies. Therefore, examining the GCX’s role in catalyzing a variety of neurodegenerative diseases beyond just AD, could prove widely beneficial. For example, GCX loss has been demonstrated in subcortical vascular dementia, a disease with notably similar vascular components to AD. In an extensive study examining the GCX’s role in neurovascular disease, Yoon et al. reported thinning of the GCX along with increased capillary stalling in a vascular dementia animal model (Yoon et al., 2022). Additionally, after enzymatic degradation of the GCX, capillary stalling increased mainly due to leukocyte plugging and an upregulation of endothelial adhesion receptors, suggesting a causal relationship between GCX and vascular dementia pathology (Yoon et al., 2022). Researchers have also investigated the use of GCX components in plasma as an early biomarker in other neurodegenerative diseases. In a mouse model of multiple sclerosis, HS levels increased before symptom onset, chondroitin sulfate increased prior to symptoms and remained elevated throughout, and HA levels spiked during peak disease severity (DellaValle et al., 2018). Elevated oxidative stress decreased NO levels, and increased glycoprotein deposits in cerebral tissue have also widely been attributed to GCX shedding in neurodegenerative diseases (Pacher et al., 2007; Bonneh-Barkay and Wiley, 2009; Siebert et al., 2014). Although an acute event, ischemic stroke events exhibit similar GCX-related neurovascular pathologies. Ischemic stroke victims display loss of the GCX corresponding with increased neuronal and vascular inflammation (Ko et al., 2020). The initial blockage of blood flow and subsequent reperfusion initiates a rise of ROS followed by matrix metalloproteinases (MMPs) and other proteolytic enzymes which then shed the GCX (Yu et al., 2019). There is substantial evidence of increased levels of shed GCX components like HS and syndecan in the plasma of stroke victims (Rehm et al., 2007; DellaValle et al., 2019). The deteriorated extracellular GCX structure allows for direct interaction between immune cells and the endothelium, eventually exacerbating neurological impairment (Mockl, 2020; Bettcher et al., 2021a; Bettcher et al., 2021b; Liu et al., 2023). Another acute event that occurs upstream of neurodegenerative diseases, traumatic brain injury, is also known to induce a GCX-related neuropathological cascade (Gonzalez Rodriguez et al., 2018; Zou et al., 2021; Anand et al., 2023). Increased shedding of GCX components after traumatic brain injury is associated with a negative survival rate, implicating the GCX’s role in pathology. After injury, an upregulation of ROS, immune cell adhesion, and sheddases results in BBB hyperpermeability, neuroinflammation, and leukocyte attachment (Zou et al., 2021).

Lastly, the process of aging is worthy of mention due to age being the number one risk factor for the majority of neurodegenerative diseases (Hou et al., 2019) and known to be associated with a diminished GCX (Machin et al., 2018; Majerczak et al., 2019). Machin et al. proposed the hypothesis that age-related GCX shedding precedes vascular dysfunction, ultimately promoting a variety of cardiovascular and neurological disease pathologies (Machin et al., 2019).

In summary, it is still emerging whether GCX shedding precedes AD and other neurological disorders characterized by vascular abnormalities. The early evidence described here indicates a correlation between GCX integrity and disease severity.

### 3.2 Proposed mechanism of endothelial glycocalyx shedding in Alzheimer’s disease

**FIGURE 7:**
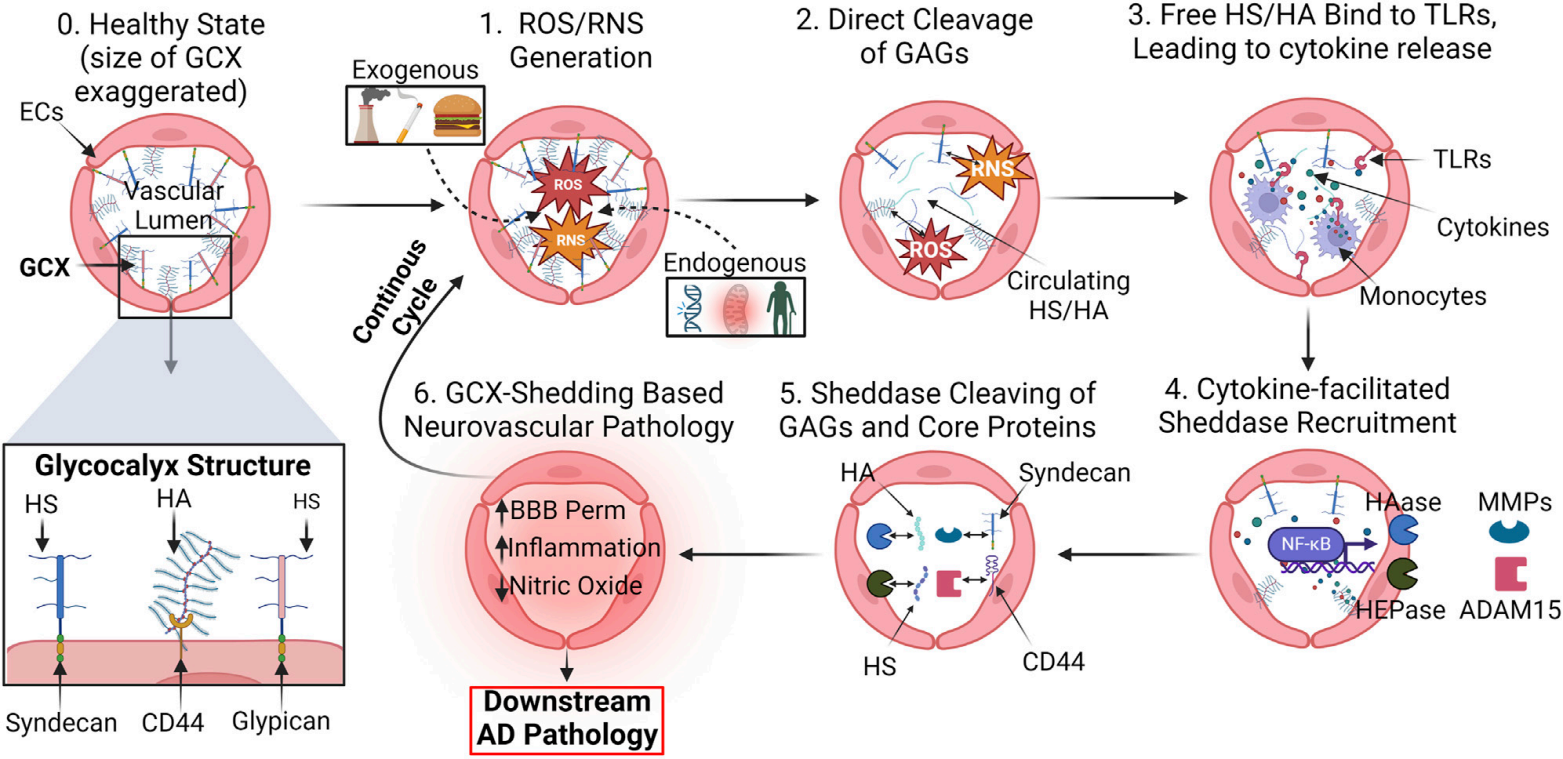
Proposed mechanism of GCX degradation resulting in neurovascular pathology: (0.) In a healthy state, the GCX robustly lines the surface of endothelial cells to regulate physiology. A zoomed in view of key GCX components is also shown: Syndecans and glypicans bind heparan sulfate (HS) and CD44 binds hyaluronic acid (HA). The proposed mechanism of degradation is as follows: (1.) ROS/RNS upregulation in cerebral vasculature due to a variety of endogenous and exogenous factors. (2.) ROS/RNS directly cleave key GCX GAGs HS and HA from the endothelium. (3.) Circulating HS and HA bind to toll-like-receptors (TLRs) on endothelial and immune cells, facilitating the upregulation of cytokines. (4.) GCX sheddases heparinase (HEPase) and hyaluronidase (HAase), as well as cleavers of core proteins like matrix metalloproteinases (MMPs) and ADAM15, are recruited. (5.) Sheddases continue to deteriorate the GCX. (6.) With a compromised GCX, vascular permeability, inflammation, and impairment of mechanotransduction-dependent responses like NO production ensue, leading to eventual AD pathology and a continuous, ferocious cycle of endothelial dysfunction (van Golen et al., 2014; Qu et al., 2021; Zhao et al., 2021). Created with BioRender.com.

The means by which the GCX is shed during AD pathology remains a topic of intense investigation. In physiology, GCX homeostasis is meticulously maintained through a dynamic interplay of synthesis, protection, and shear stress sensing mechanisms. Endothelial cells continually synthesize and assemble GCX components, while physiological levels of shear stress from blood flow reinforce its structure and stimulate GAG synthesis pathways (Alphonsus and Rodseth, 2014; Banerjee et al., 2021). Endogenous factors like NO, prostacyclin, and angiopoietin-1 interact with the GCX to regulate vascular tone, permeability, and stability (Becker et al., 2010; Potje et al., 2020; Qiu et al., 2022). Additionally, antioxidants such as superoxide dismutase help counteract oxidative stress-induced GCX damage, ensuring its integrity and proper function (Beresewicz et al., 1998). However, in pathology, numerous mediators, often interrelated, facilitate GCX degradation, directing a persistent cycle of endothelial dysfunction. These mediators include, but are not limited to ROS/RNS, inflammatory cytokines, sheddases, and MMPs (Masola et al., 2021; Zhao et al., 2021). Thus, understanding the mechanisms underlying the GCX dysregulation cycle is crucial for developing targeted therapeutic interventions to preserve vascular health and potentially prevent downstream neurological dysfunction (Figure 7).

ROS and RNS play a role in direct cleavage of GAGs, as well as the recruitment of other harmful compounds. Three principal reactive species that directly sever HA and HS GAG chains from core protein binding sites include hydroxyl radicals (•OH), carbonate radical anions (CO3•−), and hypochlorous acid (HOCl) (van Golen et al., 2014). Generation of these reactive species stems from multiple sources but one of the most pertinent endogenous routes involves their secretion by immune cells and the destabilization of the synthase that produces counteractive NO (Alderton et al., 2001; Forman and Torres, 2002). Other sources of ROS/RNS production may stem from the weakening of free radical neutralizing systems due to aging, as well as environmental factors such as a high-fat diet, air pollution, and smoking (Pérez et al., 2009; Di Meo et al., 2016). ROS/RNS production sets off a cascade of vascular pathologies, among which cleavage of the GCX is a notable factor.

Upon initial cleavage of the HA and HS GCX chains, a cyclic inflammatory cascade promotes further GCX shedding (Figure 7). Circulating HS and low molecular weight HA bind to toll-like receptors on the surface of macrophages and endothelial cells, facilitating the release of inflammatory cytokines such as tumor necrosis factor-α (TNF-α), interleukin-1β (IL-1β), and IL-8 (Johnson et al., 2002; Taylor et al., 2004; Scheibner et al., 2006; Qu et al., 2021). In conjunction with ROS/RNS, cytokines recruit a variety of sheddases to the endothelial cell surface including MMPs, hyaluronidase, and heparinase (Dempsey et al., 2000; Monzon et al., 2010; Ramnath et al., 2014). These enzymes not only have the capacity to further shed GAGs but also threaten GAG-anchoring core proteins in the transmembrane domain of the cell. For example, MMP1, MMP9, and MMP14 remove the HS proteoglycan core proteins syndecan-1 and syndecan-4, respectively, while MMP9 also cleaves chondroitin sulfate (Endo et al., 2003; Sieve et al., 2018). A disintegrin and metalloproteinase 15 (ADAM15) is responsible for the scission of CD44, the core protein responsible for HA binding to the endothelial cell surface (Yang et al., 2018). Additionally, heparinases and hyaluronidases continuously remove HS and HA, further complicating GCX recovery attempts by the cell.

Understanding how the GCX degradation cascade is initiated and translating the knowledge to the development of potential avenues for reversal of GCX degradation could prove critical to the amelioration of a plethora of vascular-related cognitive diseases, including AD. Several mediators of GCX dysfunction are found at elevated levels in patients suffering from AD. Excessive generation of GCX cleavers ROS and RNS has been linked to the pathogenesis of AD, as well as variety of age-related neurodegenerative diseases (Manoharan et al., 2016). Notably, elevated levels of ROS production are found to precede late-stage AD events like Aβ deposits and NFTs, further supporting the notion that vascular dysfunction propagates late-state AD events (Ahmad et al., 2017). Furthermore, postmortem AD brain tissues show an increase in cytokines IL-1β and IL-6 (Griffin et al., 1989; Hull et al., 1996; Zheng et al., 2016), which suggests both the presence of excess circulating GCX components as well as increased immune cell infiltration and systemic inflammation processes occurring throughout disease progression. MMP-9 was also found at significantly elevated levels in the plasma of AD patients compared to controls (Lorenzl et al., 2003). Although the abovementioned mediators of GCX shedding serve diverse functions, establishing their involvement in the pathophysiology of AD supports the hypothesis of GCX-dependent contributions to the diseases.

## 4 Current challenges

### 4.1 Imaging and quantifying cerebrovascular glycocalyx and cerebral vascular changes

To investigate the potential role of GCX integrity in neurodegenerative disease progression, it is crucial to improve both *in vitro* and *in vivo* imaging techniques of the cerebrovascular GCX. Currently, the quantification of GCX components such as HS, HA, and syndecan-1 in plasma is the primary method of assessing GCX shedding. However, additional, more direct approaches should be considered to evaluate the cerebrovascular GCX structure in preclinical, clinical, and postmortem AD tissue.

Transmission electron microscopy (TEM) is a principal technique for imaging the GCX in fixed, preserved conditions. However, one of the main challenges in TEM is preserving the sample in a way that accurately reflects the GCX ultrastructure (Haeren et al., 2016). Traditional preparation processes typically involve dehydration of the sample, causing the GCX to collapse in on itself and drastically altering its organization and thickness (Hempel et al., 2020). To address this issue, rapid freezing/freeze substitution (RF/FS) has emerged as a promising technique for sample preparation. Ebong et al. demonstrated that using RF/FS, the GCX of bovine aortic endothelial cells measured an average of 11 μm, compared to an insubstantial 0.040 μm using traditional TEM (Ebong et al., 2011). Despite the promise of RF/FS in accurately preserving the GCX, it remains a labor-intensive, delicate process that is unable to image specific GCX components.

To address the limitations of traditional TEM, confocal laser scanning microscopy (CLSM) is a promising method that can fluorescently label specific GCX components. While the characterization of GCX GAGs remains a challenge due to their high degree of heterogeneity and lack of antigenicity, innovative antibodies and lectins (such as wheat germ agglutinin) can be utilized to tag domains of GAG components of the GCX, for fluorescent labeling and subsequent CLSM visualization (Mockl, 2020). By combining RF/FS with immunolabeling-based CLSM, Twamley et al. were able to analyze the morphology of specific GCX components (Twamley et al., 2021), advancing the state-of-the-art in labeling and quantification of the GCX.

Despite the recent advances of *in vitro* techniques, development of unique solutions to microscopic imaging of the cerebrovascular GCX *in vivo* in functional tissue remains essential for elucidating the nature and function of the structure in the body. Two-photon light scanning microscopy (TPLSM) utilizes dual-photon absorption to increase penetration depth and allow for fluorescent imaging of living tissue (Denk et al., 1990). By contrasting fluorescent dextran in the lumen with wheat germ agglutinin-tagged GCX, TPLSM has been used to assess GCX thickness, BBB permeability, and vascular geometry (Yoon et al., 2017; Kucharz et al., 2022). Results indicate that the use of *in vivo* TPLSM leads to a more robust GCX than what is observed *ex vivo* and offers a functional approach to assessing GCX thickness, BBB permeability, and vascular geometry. However, this imaging technique will likely be limited to pre-clinical animal studies for cerebrovascular GCX imaging due to its invasiveness, high cost, slow imaging speed, and the need for fluorescent contrast agents.

Another option for *in vivo* assessment of the GCX that demonstrates more clinical potential is side-stream dark field imaging (SDF). SDF measures the degree of penetration of red blood cells into the GCX through a reflected LED light. Based on the extent of penetration, computer programs like Glycocheck can correlate the perfused boundary region (the permeable part of the GCX) to a GCX thickness (Iba and Levy, 2019). A recent protocol developed by Haeren et al. was the first to utilize SDF of the cerebrovascular GCX in a clinical setting. Based on the study, SDF could effectively quantify GCX thickness in the cerebral vasculature, was straightforward to operate, and safe for the use on human patients (Haeren et al., 2018). SDF and Glycocheck offer the advantage of intraoperative measurement of GCX thickness without the need for fluorescent dyes, long processing times, and image acquisition expertise (Haeren et al., 2017; Berhouma et al., 2020). However, SDF and Glycocheck still pose several drawbacks due to indirect GCX measurement and may be affected by motion-induced blurring. With the applicability of SDF and Glycocheck, an increasing amount of data on cerebrovascular GCX integrity in neurodegenerative disease is expected to emerge in the coming years, offering valuable insights for tailored pre-clinical and clinical applications.

Finally, due to the substantial difficulties of directly quantifying the GCX *in vivo*, novel, translational imaging modalities which can broadly quantify vascular changes will be instrumental in elucidating the causal role, if any, of GCX impairment on AD progression during longitudinal studies. Some studies have utilized dynamic contrast-enhanced magnetic resonance imaging (DCE MRI) to investigate BBB leakage in early AD (Figure 1) (van de Haar et al., 2016; Moon et al., 2023). However, DCE MRI has limitations in studying BBB permeability primarily due to its inability to distinguish between blood flow and permeability changes accurately (Manning et al., 2021). To overcome this, a promising approach is to utilize quantitative ultra-short time-to-echo contrast-enhanced magnetic resonance imaging (QUTE-CE MRI) to holistically quantify BBB permeability changes over time more accurately. QUTE-CE MRI offers a safe, accurate, and non-invasive method of quantifying BBB leakage making it an ideal *in vivo* quantitative method for longitudinal studies (Gharagouzloo et al., 2015; Leaston et al., 2021a; Leaston et al., 2021b). Furthermore, QUTE-CE MRI’s precise quantification of BBB changes facilitates targeted investigation into key regions affected in AD—like the hippocampus, thalamus, amygdala, and cerebral cortex, while still encompassing the entire brain in the study (Kapasi and Schneider, 2016; Nelson et al., 2016). Compared with traditional MRI, QUTE-CE MRI allows for more precise measurement of BBB permeability due to its ability to capture ultra-short echo times, which are sensitive to subtle changes in tissue microstructure and permeability. This enables researchers and clinicians to obtain more accurate and reliable data on BBB integrity, facilitating the possibility of prolonged longitudinal studies on a key read out for neurovascular health. With the continued development of these revolutionary imaging methods, unprecedented insight into GCX integrity, dynamics of BBB permeability, and overall cerebral vascular health are positioned to be elucidated, heralding a transformative era in vascular and AD research.

### 4.2 *In Vitro* modeling of the BBB

Another substantial challenge with elucidating the GCX’s role in AD involves the development of suitable *in vitro* models of the BBB. Access to preclinical and clinical *in vivo* settings that incorporate the complex nature of the BBB is limited. Therefore, the development of accurate yet high throughput *in vitro* models of the BBB structure remain indispensable to the field for their reproducibility, cost-effectiveness, and accessibility. *In vitro* BBB models which can accurately reproduce the interplay between the GCX, barrier function, and pathophysiology are critical to examining the GCX-vascular hypothesis of AD. Despite significant research in this area, recent models vary widely in their fabrication methods, flow conditions, model geometry, applicability, and versatility (Table 1). Developing an *in vitro* system which balances these parameters to fit the experimental goals continues to be a priority.

**TABLE 1:** Choosing the Right BBB Model: Key Considerations and Comparative Analysis: This table offers a thorough evaluation of recent BBB models, examining each model against five pivotal factors essential for achieving physiological relevance as well as experimental feasibility. “Ease of fabrication” assesses the simplicity and technical expertise required for model construction. Consideration is given to the length of cell culture required for each model, impacting experimental throughput as well. “Physiological Flow” evaluates the model’s ability to simulate hemodynamic flow conditions experienced by cerebral blood vessels. “Physiological geometry” examines how closely the model’s structure resembles the innate architecture of the neurovasculature. “Post-experimental Analysis” assesses the ease and effectiveness of conducting post-experimental biological assays including barrier functionality, protein quantification, and fluorescent microscopy. “Model Versatility” is determined by the model’s capability to adapt to different mechanical environments, culture conditions, and experimental factors, enabling diverse applications. Based on this assessment, a millifluidic, transwell-compatible model shows promise for future *in vitro* studies exploring the role of the GCX in AD.

Model type	Graphical representation	Ease of fabrication	Physiological flow	Physiological geometry	Post- experimental analysis	Versatility	Ref
Millifluidic Transwell (Ebong Model)	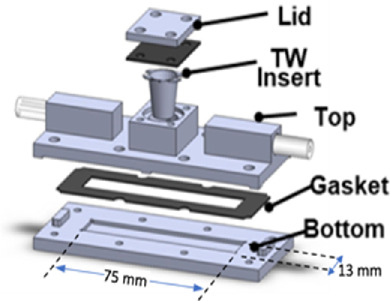	✓	✓		✓	✓	Harding et al. (2022)
Transwell	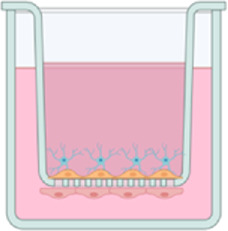	✓			✓		Ito et al. (2019), Li et al. (2019)
Organoid	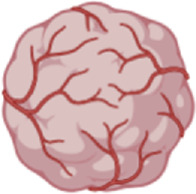	**?**		✓	✓		Cho et al. (2017b), Berhouma et al. (2020)
Organ-on-a-Chip	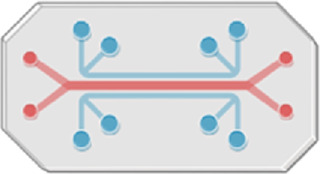		✓		✓	✓	Brown et al. (2015), Boghdeh et al. (2022)
3D Vessel	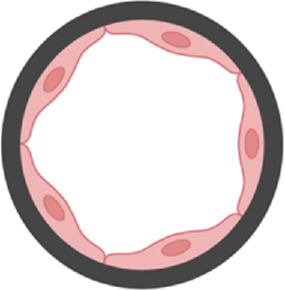		✓	✓	**?**	**?**	Marino et al. (2021), Yan et al. (2023)

Today, the field largely agrees that the primitive *in vitro* BBB model consisting of a static, brain microvascular endothelial cell monolayer cultured in a transwell insert falls short of the needed complexity to be considered a viable candidate to study GCX-BBB-AD interactions (Table 1). A primary shortcoming of this model is the lack of physiological shear stress the cells are subjected to. Hemodynamic flow conditions are increasingly recognized as essential for capturing the intricate microenvironment of the NVU *in vitro*, as endothelial cells respond dynamically to shear stress (Williams-Medina et al., 2020). Under shear stress, brain endothelial cell phenotype alterations occur, with flow models demonstrating decreased BBB permeability compared to static cultures (Santaguida et al., 2006; Cucullo et al., 2011). In specific relation to the GCX, thickness of the structure in flow conditions has been reported to increase 70%–80% compared to static conditions and approach thicknesses observed *in vivo* (Ueda et al., 2004; Tsvirkun et al., 2017; Harding et al., 2019b). Selecting a model which generates a robust GCX is essential for future studies aimed at investigating the GCX role in deterring AD pathology.

Similar to the importance of flow conditions, *in vitro* BBB models which can incorporate many cell types to recreate the complex multicellular interface are essential to accurately studying AD pathogenesis. Different cells, including primary human endothelial cells, induced pluripotent stem cell-derived (iPSC) endothelial cells, animal-derived endothelial cells, and immortalized endothelial cells, mimic the cellular lining of the brain vascular lumen (He et al., 2014; Destefano et al., 2018). Adding another layer of complexity, supportive cells such as astrocytes, pericytes, microglia, and neurons are also often incorporated and deemed essential to achieving a functional BBB model, depending on the purpose (Daneman and Prat, 2015). These diverse cell types enable the study of neurovascular function in healthy conditions, neurovascular function in neuroinflammatory and neurodegenerative conditions, therapeutic targets, and drug transport across the BBB, but it is still unclear the exact importance of them to a functional model.

In terms of fabricating a model which captures the innate geometric properties of the BBB, several innovative fabrication methods are being investigated (Table 1). Certain microfluidic systems utilize channel culturing techniques to replicate 3D blood vessels and allow for physiological flow and cell-cell interactions, providing increased physiological relevance (Bonakdar et al., 2017; Hajal et al., 2022). However, these systems can be technically complex to fabricate and operate, requiring specialized expertise and equipment. Scaffold-based approaches create three-dimensional frameworks using porous membranes or hydrogels, promoting cell-cell interactions and barrier formation but may lack the ability to precisely control spatial organization and have limited scalability (Marino et al., 2021). Novel 3D organoid models enable *in vivo* like formations of complex BBB structures (Cho CF. et al., 2017; Bergmann et al., 2018; van Dyck et al., 2023). However, organoid culturing techniques are still under development, and challenges remain in achieving reproducibility, scalability, and mechanically accurate microenvironments. The preferred option for fabrication method often depends on research expertise, experimental design, and throughput considerations.

With the goal of taking all of these factors into consideration, our lab has developed a custom-made millifluidic device that is compatible with standard transwell inserts to develop a physiologically relevant study while maintaining high throughput for analysis of the role of the GCX in AD (Table 1) (Harding et al., 2022). This design combines standard transwell inserts with a microfluidic device featuring validated shear stress profiles. It also incorporates primary human brain endothelial cells, pericytes, and astrocytes, enhancing fidelity for studying BBB dynamics and neurological consequences of GCX impairment. The advantages of this model include its straightforward and reusable fabrication via 3D printing and insertion of transwell inserts, physiological flow rate, robust post-experimental analysis, and versatility with the ability to culture different cell types and flow rate changes. Although there are more physiological fabrication approaches which achieve 3D geometries and direct cell-cell contact, this transwell system is comparable to previous studies (Table 1). Initial studies demonstrate that GCX thickness and tight junction expression increase in tri-cultures containing endothelial cells, astrocytes, and pericytes. Furthermore, exposure to 12 dyne/cm^2^ of shear stress increased GCX coverage, potentially supporting the lower observed permeability in flow conditions (Harding et al., 2022). This model is well suited for balancing the needed physiological relevance with ease of use required to study the GCX’s role in AD.

### 4.3 Experimental methods of studying GCX-related Alzheimer’s disease pathology

Due to the GCX’s heterogeneity and complex functionality to the NVU, devising experimental strategies to study the relationship between GCX degradation and vascular-related AD pathology is a critical consideration. Traditionally, the GCX is degraded through enzymatic cleavage of its GAGs (McDonald et al., 2016; Yalcin et al., 2018). Although this technique may be sufficient for other vascular-organ interfaces, the multicellular, dynamic nature of the BBB may lead to the enzymes having several off-target effects on brain cell function, resulting in questions of causation during downstream biological analysis (Yang et al., 2021; Zhang et al., 2021).

For this reason, recent gene editing advancements offer practical means to investigate the GCX’s role in AD-vascular pathology (Reitsma et al., 2007). Techniques like CRISPR/Cas9, shRNA, and siRNA effectively knockdown or knockout GCX core protein expression, facilitating the study of their functional significance (Song and Yang, 2010; Abrahimi et al., 2015). By employing these techniques *in vitro* and *in vivo*, researchers can investigate the functional consequences of depleting GCX core protein expression on cellular processes i.e., barrier integrity, inflammation control, and responsiveness to the extracellular environment. While effective *in vitro*, utilizing *in vivo* knockout models which broadly target GCX core proteins presents challenges due to their expression in cell types beyond endothelial cells, increasing the risk of off-target effects (Couchman and Pataki, 2012). To address this issue, efforts should be directed towards generating conditional transgenic animal models with endothelial cell-specific GCX core protein knockouts (Liao et al., 2001; Fahs et al., 2014). These genomic editing approaches would contribute to a deeper understanding of the roles played by GCX core proteins in disease processes, including neurodegenerative disorders like AD.

The impact of GCX-specific knockout mice will be powerful when combined with the *in vivo* imaging techniques described in Section 4.1. For example, *in vivo* imaging techniques such as TPLSM offer the potential to track the neurovasculature and GCX over time with greater depth and precision compared to traditional techniques (Haeren et al., 2016; Kilic et al., 2020). Additionally, the use of QUTE-CE MRI provides a promising method to track neurovascular abnormalities, BBB permeability, cerebral blood flow, and neuroinflammation over the course of disease progression in a non-invasive, clinically-relevant way (Leaston et al., 2021a). Finally, post-mortem-tissue analysis utilizing RF/FS preservation techniques can visualize GCX thickness and coverage in a manner previously unobtainable with traditional preservatives. Given the relatively recent appreciation for GCX involvement in neurodegenerative disease and the development of these ground-breaking imaging modalities, the foundation is set to firmly establish a prospective relationship between GCX degradation and downstream AD pathology.

A legitimate concern with evaluating the direct relationship between GCX impairment and AD progression *in vitro* and *in vivo* is modelling complex disease in a relevant way. To generate AD-like pathology both *in vitro* and *in vivo*, researchers may employ various techniques to mimic key aspects of the disease. *In vitro*, AD-like pathology can be induced in cultured cells or blood-brain organoids by exposing them to Aβ peptides, tau protein aggregates, or neuroinflammatory cytokines (Sharma et al., 2021; Schreiner et al., 2022). This can lead to the formation of Aβ plaques, tau hyperphosphorylation, synaptic dysfunction, and neuronal cell death, resembling key features of AD pathology (Chen et al., 2021). *In vivo,* there are several commercially available transgenic rodent models of AD but are a constant subject of debate, primarily because of the disease’s complex etiology and the fact that rodents cannot naturally develop it (Yokoyama et al., 2022). Some commonly deployed models for studying AD which are well-characterized for inducing neuropathological features and cognitive deficits characteristic of the disease include the mutant amyloid precursor protein and presenilin 1 (APP/PS1), Tg2576, and 5xFAD lines (Gotz et al., 2018; Sanchez-Varo et al., 2022). A major advantage of these AD models is abnormal protein folding in the brain, appreciable vascular changes, and cognitive deficits witnessed and progressing between 6 and 12 months of age in most models, setting a feasible time scale for longitudinal studies of AD (Webster et al., 2014). However, one of the persistent shortcomings of these transgenic models is their neuro-centric focus and failure to exhibit likely vascular changes present in AD. However, inducing impairment of the GCX in these models and subsequently longitudinally examining the animals for variations in cognitive function would be valuable in establishing the GCX’s role as a contributor to the propagation of AD. This would be particularly significant if AD models with GCX deficiency were to exhibit exacerbated symptoms.

Moreover, beyond studying AD models, exploring the role of the GCX in AD also involves considering the aging aspect, the number one risk factor of the disease. Aged cells and rodent models provide valuable insights into the dynamic changes occurring within the GCX as the brain matures, shedding light on its relevance to AD pathology. Aged cells exhibit physiological changes and molecular alterations that mimic aspects of aging-related neurodegeneration observed in AD while also paralleling the believed thinning of the GCX with age (Ting et al., 2021; Sun et al., 2023). *In vitro*, cell lines derived from aged individuals can be used to model age-related changes in cellular function and susceptibility to AD pathology. These cells may exhibit increased oxidative stress, protein misfolding, mitochondrial dysfunction, and altered signaling pathways associated with aging and neurodegeneration (Chen et al., 2020). Likewise, investigating aged rodents *in vivo* provides insight into the effects of aging on disease progression and its potential correlation with GCX loss. Such research, considering traditional AD amyloid-based pathology, age, and GCX alterations, not only explores the potential impact of GCX loss as a major contributing factor but also potentially informs the development of novel therapeutic interventions for AD.

To address whether GCX loss exacerbates AD pathology, specific techniques can be employed. *In vitro*, GCX loss can be induced in endothelial cells as described above, followed by exposure to AD-related stimuli. *In vivo*, genetic manipulation or pharmacological approaches targeting GCX components can be combined with AD-inducing interventions in transgenic mouse models. By assessing changes in BBB integrity, Aβ clearance, neuroinflammation, and cognitive function, researchers can elucidate the role of GCX loss in worsening AD pathology and identify potential therapeutic targets for intervention.

## 5 Future work and conclusion

Neurodegenerative diseases such as AD are among the most urgent public health concerns of the 21st century, with the demand for effective therapies expected to increase alongside the aging population. Given the complex etiology of AD involving both neurological and vascular components, it is essential to advance research on the understanding of the interplay of both systems. Targeting the vascular endothelial GCX emerges as a promising avenue, offering the potential to prolong or restore critical neurovascular functions impaired in AD. Despite the ongoing uncertainty regarding whether GCX degradation precedes vascular insult and AD pathology, recent advancements in evaluating the GCX in the cerebral vasculature open doors for future work to definitively determine its viability as a therapeutic target for neurodegenerative diseases.

After conducting an extensive literature review and considering experimental feasibility, the initial approach to determining whether the GCX could serve as a viable therapeutic target for delaying, mitigating, or preventing AD symptoms involves thoroughly assessing the functionality of the GCX in relation to countering AD-related neurovascular pathologies in both *in vitro* and *in vivo* settings.

Tangible methods of accomplishing this task *in vitro* include: 1. Utilize a physiologically relevant, reliable, high degree of post-experimental analysis BBB model such as the one described by our group in a previous publication (Harding et al., 2022). 2. Use a highly transfectable shRNA knockdown technique to reduce the expression of individual GCX core proteins selectively and stably such as CD44, syndecan-1, and glypican-1 in brain microvascular endothelial cells. 3. Assess the effects of these GCX core protein knockdowns on NVU functionality using techniques such as dextran permeability assays, trans-endothelial electric resistance, and cell-cell communication. Additionally, analyzing the protein and gene expression of markers associated with AD-related neurovascular pathology to uncover potential mechanistic insights, including tight junctions, intracellular transporters, adhesion molecules, cytokines, and NO production mediators. This approach aims to elucidate the role of individual GCX components in maintaining physiological function.

*In vivo* methods of examining the role of the GCX in AD should aim to corroborate *in vitro* findings while further capturing complexities unobtainable through solely cell-based observations such as: 1. Examine distinctions among healthy mice, CD44 knockout models (Due to this knockout model’s commercial availability and minimal off-target effects), and amyloid-based AD models like APP/PS1, to understand the ramifications of GCX impairment in contrast to the traditional neurocentric manifestation of AD, particularly concerning AD-related neurovascular dysfunction (Protin et al., 1999; McKallip et al., 2002). 2. Longitudinally interrogate changes in BBB permeability using translationally relevant QUTE-CE MRI until reaching an appropriate advanced age, to consider the compounded effect of aging in relation to GCX impairment and AD progression. 3. Perform immunological analysis on brain sections and plasma samples, comparing baseline samples to those obtained after aging in the three groups, and probing for similar markers as in the *in vitro* study. These findings will explore whether the knockout of a crucial GCX core protein, CD44, triggers BBB dysfunction, while also distinguishing the effects of BBB damage stemming from abnormal protein folding linked to AD.

If the data from an initial study were to show promise, further investigations could be pursued on determining the GCX’s relevance in AD, involving more labor, cost, and time-intensive approaches as those highlighted in Section 4.3. If evidence were to mount indicating the significant involvement of the GCX in AD pathology, it becomes imperative to explore novel GCX-regenerating therapeutics in these models, assessing their potential to ameliorate the severe pathological symptoms of the disease (Banerjee et al., 2021). While significant efforts are still required to ascertain the role, if any, of this vascular-based structure in protecting against AD, recent advancements in biotechnology have undoubtedly opened the door to feasibly address this question like never before. As we continue to navigate the complexities of AD and the potential of a substantial vascular role, the pursuit of a deeper understanding of the GCX’s relationship to the disease stands as a critical step toward effective treatments and improved public health outcomes.

## Abbreviations

AD, Alzheimer’s Disease; Aβ, Amyloid Beta; BBB, Blood Brain Barrier; CLSM, Confocal Laser Scanning Microscopy; CNS, Central Nervous System; DCE MRI, Dynamic Contrast-Enhanced Magnetic Resonance Imaging; eNOS, Endothelial Nitric Oxide Synthase; GAG, Glycosaminoglycan; GCX, Endothelial Glycocalyx; HA, Hyaluronic Acid; HS, Heparan Sulfate; ICAM-1, Intercellular adhesion molecule-1; IL, Interleukin; MMP, Matrix Metalloproteinase; NFT, Neurofibrillary Tangle; NO, Nitric Oxide; NVU, Neurovascular Unit; RF/FS, Rapid Freezing/Freeze Substitution; RNS, Reactive Nitrogen Species; ROS, Reactive Oxygen Species; SDF, Side-Stream Darkfield Microscopy; TEM, Transmission Electron Microscopy; TPLSM, Two-Photon Light Scanning Microscopy; QUTE-CE MRI, Quantitative Ultra-Short Time-To-Echo Contrast-Enhanced Magnetic Resonance Imaging.
